# Supplementary material for: Association between oral microbiome and seven types of cancers in East Asian population: a two-sample Mendelian randomization analysis
Source: Front Mol Biosci. 2023 Nov 21;10:1327893. doi: 10.3389/fmolb.2023.1327893 (PMC10702768; doi:10.3389/fmolb.2023.1327893)
Supplement: Supplementary file 1 [file DataSheet1.ZIP › Supplementary Materials/MR plots of saliva/saliva═╝/Breast cancer/pheno.2222_to_breast cancer_forest.pdf]

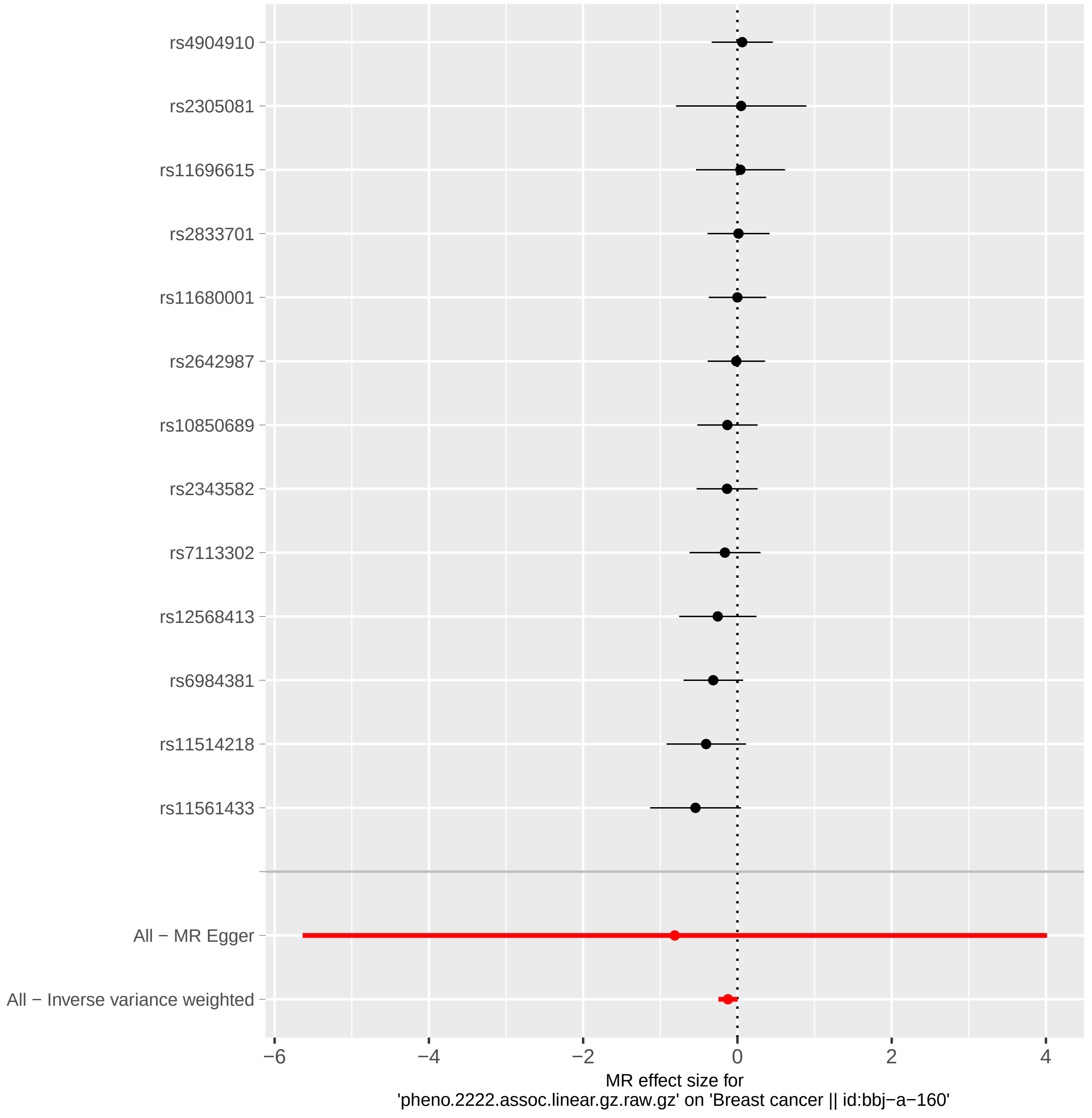

rs4904910
rs2305081
rs11696615
rs2833701
rs11680001
rs2642987
rs10850689
rs2343582
rs7113302
rs12568413
rs6984381
rs11514218
rs11561433
All − MR Egger
All − Inverse variance weighted
−6
−4
−2
0
2
4
MR effect size for
'pheno.2222.assoc.linear.gz.raw.gz' on 'Breast cancer || id:bbj−a−160'
